# Dynamic Changes in Volatile Organic Compounds and Quality Traits in Two Wheat Cultivars During Grain Filling: An Integrated Volatilomics Study Using HS-GC-IMS and HS-SPME-GC-MS

**DOI:** 10.3390/foods15152639

**Published:** 2026-07-28

**Authors:** Hongyan Mao, Lianzheng Liu, Jinshan Zhang, Li Yue, Jiamin Wang, Zulipiya Maimaiti, Yueren Xu, Tingting Zhang, Na Liu, Junmei Cao, Halidan Yikeremu, Xinzhong Zhang, Shubing Shi, Ming Yu

**Affiliations:** 1 College of Agriculture, Xinjiang Agricultural University, Urumqi 830052, China; maohongyan1226@126.com (H.M.);; 2 Institute of Crops Research, Xinjiang Academy of Agricultural Sciences, Urumqi 830091, China

**Keywords:** wheat, volatile organic compounds (VOCs), grain filling, HS-SPME-GC-MS, HS-GC-IMS, quality traits

## Abstract

This study investigated quality traits and volatile organic compounds (VOCs) in two winter wheat varieties with different gluten types (Xinliang 169, medium-strong gluten, and Xinliang 607, medium-weak gluten) across five grain-filling stages (5, 10, 20, 30, and 40 days after anthesis, DAA) under heated headspace conditions (80 °C). During grain filling, moisture and total soluble sugar (TSS) decreased significantly, whereas hardness, thousand-kernel weight (TKW), and starch content increased significantly. Protein followed a typical “high–low–high” pattern. Using headspace gas chromatography–ion mobility spectrometry (HS-GC-IMS) and headspace solid-phase microextraction gas chromatography–mass spectrometry (HS-SPME-GC-MS), 80 and 125 VOCs were detected, respectively. A total of 19 preliminarily prioritized candidate odorants (e.g., 1-penten-3-one, 2,3-butanedione, and 3-methylbutanal) were screened using orthogonal partial least squares discriminant analysis (OPLS-DA, VIP > 1) and relative odor activity value (ROAV ≥ 1). Based on literature odor descriptors, the aroma profile shifted from green, malty notes during early grain filling to waxy, fatty, and grain-like notes during late grain filling. XL169 was characterized by grassy-green, malty, and fatty grain-like notes, whereas XL607 was dominated by creamy, buttery, and sweet-fruity woody notes. Correlation analysis revealed significant correlations between high-priority candidate odorants (2,3-butanedione, (*E*)-2-octenal, and 3-hydroxy-4,5-dimethyl-2(5H)-furanone) and quality traits (starch, TSS, and protein). These exploratory findings provide a preliminary reference profile for understanding how grain filling affects wheat VOCs.

## 1. Introduction

Wheat (*Triticum aestivum* L.) is the most widely cultivated crop worldwide, providing approximately 20% of global energy and protein intake [1]. As consumer demand for high-quality wheat products rises, wheat aroma has become a key factor in purchasing decisions and sensory acceptance, underscoring the importance of understanding the dynamic changes and formation mechanisms of grain aroma [2,3]. Therefore, a thorough understanding of quality changes and the volatile organic compounds (VOCs) that develop during the grain-filling stages is essential for optimizing wheat breeding and quality regulation.

VOCs determine odor quality and correlate with harvest time, yield, seed quality, and end-use processing performance [2,4]. As a unique metabolic fingerprint, VOCs sensitively reflect internal metabolic activity, stress responses, and developmental status during grain growth [5,6,7,8]. Recently, VOC profiling has been widely used for wheat quality evaluation, geographical traceability, postharvest storage monitoring, cultivar discrimination, and sensory prediction [5,9,10,11].

Grain filling is a critical period for dry-matter accumulation and quality development [12,13]. During this stage, complex physiological and biochemical processes—including starch synthesis, lipid oxidation, amino acid metabolism, and reactions in the lipoxygenase pathway—directly drive the production, transformation, and accumulation of volatile metabolites [5,13]. Previous studies have confirmed that wheat VOC profiles are significantly influenced by genotype, ecological region, planting altitude, harvest year, storage conditions, and postharvest treatments [2,6,12,14,15]. However, most studies focus on mature grains, stored wheat, or processed products such as whole-wheat bread and pasta [4,5,16], or on single time points across multiple cultivars, leaving the dynamic trajectories of VOCs during the grain-filling period largely unexplored. Consequently, the temporal patterns and genotypic variation in VOCs during grain filling remain unclear, limiting mechanistic understanding of wheat aroma formation.

Currently, headspace solid-phase microextraction gas chromatography–mass spectrometry (HS-SPME-GC–MS) and gas chromatography–ion mobility spectrometry (GC-IMS), coupled with multivariate statistical analysis (MSA), are widely used to characterize volatile components [5,17]. These techniques have identified aldehydes, alcohols, ketones, pyrazines, furans, and esters as the major VOC classes in wheat flour, wholemeal, and processed products [2,8,9]. Previous studies have demonstrated the utility of combined GC-MS and GC-IMS approaches for identifying key aroma compounds in wheat. For instance, hexanal, benzaldehyde, nonanal, 2-pentylfuran, and (*E*)-2-octenal were identified as key aroma compounds in green wheat across treatments [17]. Additionally, 4-hydroxy-2,5-dimethyl-3(2H)-furanone, 3-hydroxy-2-methyl-4-pyrone, 2,3-butanedione, 1-furfuryl pyrrole, furfuryl alcohol, and 2-acetyl-1-pyrroline were identified as key contributors to the liking of whole-wheat bread aroma [9]. Furthermore, the volatile profile of wheat grains could serve as an indicator of seed viability [4], classification [15,18], pest infection [5], and grain spoilage [19]. Despite the proven value of dual-platform approaches for mature grains or processed products, few studies have combined HS-SPME-GC-MS and HS-GC-IMS to elucidate VOC dynamics and genotypic variation across multiple grain-filling stages. Moreover, the correlation between VOCs and primary quality traits during grain filling remains poorly understood.

Accordingly, this study investigated dynamic changes in quality traits and VOC profiles across five grain-filling stages in two wheat cultivars (strong-gluten vs. weak-gluten) using a dual-platform volatilomics approach combining GC-IMS and GC-MS. This complementary approach provides broad coverage of small-molecule markers and diverse chemical classes, which neither platform alone can achieve. The objectives were to (1) characterize temporal changes in kernel quality parameters in two contrasting gluten types; (2) identify stage- and genotype-specific VOCs and priority candidate odorants; (3) reveal the succession of VOC profiles during grain filling across quality types; and (4) establish correlations between quality traits and priority VOCs to elucidate the relationship. This five-stage, two-cultivar design enables observation of VOC dynamics during grain filling and their temporal correlation with quality-trait development, providing a useful reference for flavor-oriented germplasm evaluation and quality management.

## 2. Materials and Methods

### 2.1. Plant Material and Sampling

Field experiments were conducted during the 2025 growing season at the Anningqu Experimental Station of the Xinjiang Academy of Agricultural Sciences, in a typical irrigated agricultural region in northern Xinjiang, China. Two winter wheat cultivars were used: Xinliang 169 (XL169, medium-strong gluten) and Xinliang 607 (XL607, medium-weak gluten), provided by the North Xinjiang Winter Wheat Breeding Innovation Team. The experiment used a randomized complete block design with three replicates. Each plot measured 12 m^2^ (6 m × 2 m) and was manually sown at a seeding rate of 300 kg ha^−1^. Nitrogen fertilizer (urea, ≥46% N) was applied at 330 kg N ha^−1^. All treatments received equal amounts of phosphorus and potassium fertilizers, 120 kg P_2_O_5_ ha^−1^ and 90 kg K_2_O ha^−1^, applied as a single pre-rotary-tillage dose. Irrigation was applied at 3150 m^3^·ha^−1^ during the growing period. Field management followed standard local practices. Grains were sampled at five developmental stages: 5, 10, 20, 30, and 40 days after anthesis (DAA). For each cultivar and stage, approximately 50 spikes were randomly collected from three replicate plots. Grains were pooled, manually threshed, immediately frozen in liquid nitrogen, and then stored at −80 °C until analysis.

### 2.2. Determination of Quality Traits

Wheat kernels were enzyme-inactivated at 105 °C for 30 min, then dried at 80 °C to constant weight. Thousand-kernel weight (TKW) and moisture content were calculated. Kernel hardness was measured on fresh grain using a TMS-PRO texture analyzer (Food Technology Corporation, Sterling, VA, USA) equipped with a P/50 cylindrical compression probe and a 500 N load cell. Dried samples were ground and sieved through a 0.5 mm sieve. Starch and total soluble sugar (TSS) contents were determined by the anthrone–sulfuric acid method. Soluble protein content was measured by the Biuret method. Starch, TSS, TKW, and soluble protein were reported on a dry-weight basis (DW). All analyses were performed in triplicate.

### 2.3. VOC Analysis by HS-SPME-GC-MS

HS-SPME-GC-MS was based on the literature [20], with minor modifications. VOCs were analyzed using a GC-MS system (GCMS-QP2030, Shimadzu, Kyoto, Japan) equipped with a DB-WAX column (30 m × 0.25 mm × 0.25 µm) and an autosampler (CTC-PAL, CTC Analytics AG, Zwingen, Switzerland). A 50/30 µm DVB/CAR/PDMS SPME fiber (Supelco, Bellefonte, PA, USA) was used for headspace extraction. A 1.5 g sample of wheat grain was weighed into a 20 mL headspace vial, and 0.3 g of NaCl was added to improve extraction efficiency. The vial was sealed and placed on the autosampler tray for headspace extraction. The sample was equilibrated at 80 °C for 15 min, after which the conditioned fiber was exposed to the headspace at 80 °C for 45 min with shaking at 500 rpm. After extraction, the fiber was immediately inserted into the GC injector and desorbed for 5 min at 250 °C in splitless mode. The oven temperature program was as follows: initially held at 40 °C for 3 min, then raised to 120 °C at 5 °C/min, to 230 °C at 10 °C/min, and finally held at 230 °C for 10 min. High-purity helium (≥99.999%) served as the carrier gas at a constant flow rate of 1.0 mL/min. Mass spectrometry was performed using electron ionization (EI) at 70 eV. The ion source temperature was set to 230 °C, and the transfer line temperature to 280 °C. Data were acquired in full-scan mode over the *m*/*z* range 35–400.

A procedural blank was injected regularly to monitor background contamination. Peaks consistently present in blanks, along with those identified as column bleed or phthalate contaminants, were excluded from statistical analysis. The acquired mass spectra were matched against the NIST20 standard mass spectral library, and compounds with a similarity greater than 80 were considered putatively identified. For further confirmation, experimentally determined retention indices (RI) were calculated using a series of n-alkanes (C9–C33) and compared with RI values reported in the literature. Based on the combined evidence, the identified compounds were classified by confidence level. The relative contents of the individual volatile compounds were then determined using the peak-area normalization method.

### 2.4. VOC Analysis by HS-GC-IMS Analysis

The VOCs in wheat samples were analyzed via a GC-IMS device (FlavourSpec^®^, G.A.S., Dortmund, Germany) integrated with an autosampler and a headspace sampling unit. The GC was equipped with an FS-SE-54-CB-1 capillary column (15 m × 0.53 mm × 1 µm). The method was based on previous literature [21] with minor modifications. For each treatment at each sampling time, a 1.5 g sample was weighed into a 20 mL headspace vial and sealed with a PTFE/silicon septum. The detection process consisted of the following steps: After incubation at 80 °C for 20 min with shaking at 500 rpm, 500 μL of the headspace was introduced into the GC-IMS system via a heated syringe (90 °C) through a heated injection port. Nitrogen (N2, purity ≥ 99.99%) served as both the carrier and drift gas. The nitrogen flow rate was set to 2 mL/min for 2 min, then 10 mL/min for 10 min, and finally 100 mL/min for 15 min. Each biological replicate was measured in triplicate. The retention index (RI) for each VOC was determined using a series of n-ketones (C4–C9) as external reference standards. VOCs were identified by comparing RI and drift time (DT) with the GC-IMS library.

### 2.5. Relative Content Calculation

Relative quantification was performed using peak-area normalization, in which the relative content of each compound was expressed as its peak area as a percentage of the total peak area across all detected compounds [22]. The relative content was calculated using the following formula:

$$C_{i}=\frac{A_{i}}{A_{1}+A_{2}+\cdots +A_{n}}\times 100\%$$

*C_i_* is the relative content of an identified component, *A_i_* is its peak area, and *n* is the total number of identified components.

### 2.6. Relative Odor Activity Value (ROAV) Analysis

ROAV was used as a semi-quantitative tool to preliminarily prioritize volatile compounds based on their potential odor significance in the wheat samples. The ROAV of each compound was normalized to a maximum of 100, with higher ROAVs indicating a greater theoretical contribution to the overall aroma profile. Compounds with ROAV ≥ 1 were considered candidate odorants and given priority for further investigation, whereas those with 0.1 ≤ ROAV < 1 were considered to have possible modifying effects on the odor. The ROAV was calculated using the following formula:

$$ROAV=\frac{C_{i}}{T_{i}}\times \frac{T_{max}}{C_{max}}\times 100$$

where *C_i_* and *T_i_* are the relative content and the threshold for compound *i*, *T_max_*/*C_max_* is the maximum of *T_i_*/*C_i_* across all compounds in the sample, and the *C*/*T* ratio (relative content-to-threshold ratio) is the odor activity value. Odor thresholds in air were obtained from the relevant literature [23].

### 2.7. Statistical Analysis

For data visualization and descriptive presentation, VOC abundance values were cube-root transformed to reduce skewness. All measurements were performed on three technical aliquots from the pooled composite sample for each cultivar × stage combination; the data are presented as means ± the range across these technical replicates. Because biological replicates were pooled prior to analysis, no inferential statistics (e.g., ANOVA or post hoc tests) were applied. The results are interpreted descriptively, as an exploratory pilot dataset. Principal component analysis (PCA) and orthogonal partial least squares-discriminant analysis (OPLS-DA) were performed using SIMCA software (version 14.1, Sartorius Stedim Biotech, Umeå, Sweden) to assess similarities and differences among samples. Heatmap and Pearson correlation analyses were conducted using the online tools at https://www.omicstudio.cn (accessed on 20 July 2026). Bar charts were prepared in Origin 2021 (Origin Lab Corporation, Northampton, MA, USA).

## 3. Results

### 3.1. Dynamic Changes in Quality Traits During Grain Filling of Wheat

**Figure 1:**
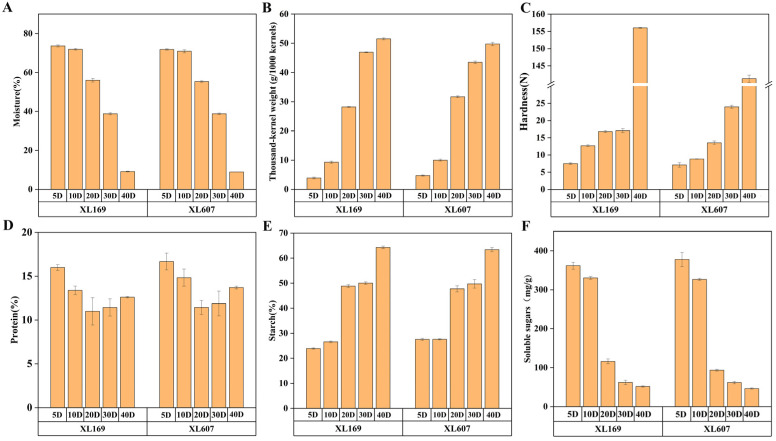
Dynamic changes in quality traits of XL169 and XL607 across five grain-filling stages (5, 10, 20, 30, and 40 days after anthesis, DAA). (**A**): moisture, (**B**): thousand-kernel weight (TKW), (**C**): hardness, (**D**): protein, (**E**): starch, and (**F**): total soluble sugar (TSS).

From 5 to 40 days after anthesis (DAA), thousand-kernel weight (TKW), kernel hardness, and starch content increased significantly in both XL169 and XL607, whereas moisture and total soluble sugar (TSS) decreased progressively (Figure 1). Grain protein content followed a typical ‘high–low–high’ pattern throughout the grain-filling period, consistent with previous observations [24]. Amino acids provide abundant precursors for cereal aroma formation, and protein content and secondary structure of proteins can affect interactions between flavor compounds and proteins, thereby influencing flavor release [2]. Moreover, wheat exhibits a close coordination between moisture and dry matter content during grain filling, and the maximum moisture content is recognized as a reliable indicator of maximum grain volume and final grain weight [13]. In agreement with this, XL169 had a higher moisture content than XL607 at the beginning of grain filling, and its thousand-grain weight also exceeded that of XL607 in later stages. TSS serves as the substrate for starch synthesis, and its concentration is closely related to starch content and positively correlated with the rate of starch accumulation [25]. This is consistent with our research, which shows that increased starch accumulation during grain filling is accompanied by a decline in soluble sugar content.

As grain filling progressed, both cultivars showed similar trends in quality traits, but with distinct genotypic differences. XL169 achieved higher final starch content than XL607, indicating more efficient starch synthesis and a longer period of active accumulation, both of which contributed to its higher final kernel dry weight [26]. Conversely, XL607 consistently maintained higher protein levels than XL169. Studies have shown that genotypic differences in grain protein content are associated with post-anthesis nitrogen uptake capacity and the efficiency of nitrogen remobilization from vegetative organs to the grain [27]. This provides more amino acid precursors for Strecker aldehyde formation [28], thereby favoring the development of a malty aroma. Total soluble sugar peaked early, then declined as carbohydrates were diverted into starch biosynthesis, potentially limiting the availability of reducing sugars for late-stage Maillard reactions that produce sweet and caramel-like aromas [29]. Notably, in addition to contributing to wheat aroma formation, nutrients such as proteins and starches also interact with VOCs, thereby influencing the release and perception of aroma [29].

### 3.2. GC-IMS Analysis of Volatile Compounds in Two Wheat Varieties

#### 3.2.1. Identification of VOCs by GC-IMS

**Figure 2:**
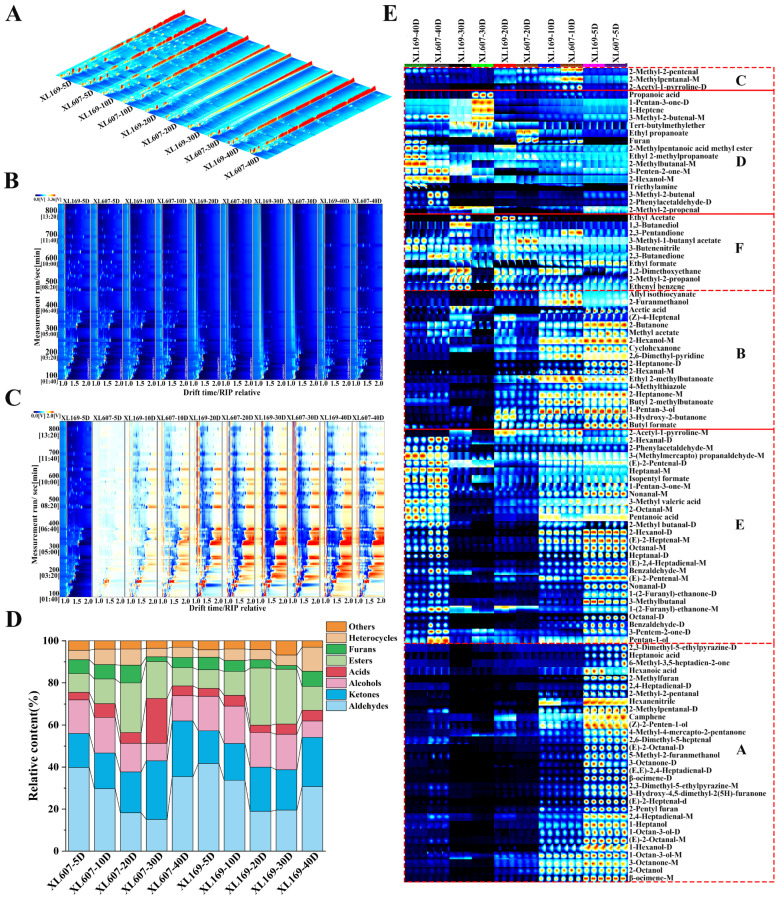
HS-GC-IMS imaging of volatile compounds from two wheat cultivars at different grain-filling stages (5, 10, 20, 30, 40 DAA). (**A**): Three-dimensional topographic spectrum. (**B**): Two-dimensional top-view plot. (**C**): Difference plot obtained by subtracting the GC–IMS spectrum of the control sample from that of the treated sample. (**D**): Relative contents of volatile compounds. (**E**): Gallery plot comparison of volatile compounds, regions A–F correspond to characteristic volatile signal areas across grain-filling stages.

To comprehensively evaluate the volatile flavor profiles of the two wheat varieties across grain-filling stages, VOCs were analyzed using HS-GC-IMS. Compounds were identified using the NIST and IMS databases based on retention index (RI) and drift time (DT). The three-dimensional topographic spectrum and the two-dimensional top view of VOCs are shown in Figure 2A and Figure 2B, respectively. Each spot on the map represents a compound, with its position determined by relative retention and drift times and its color intensity reflecting the signal amplitude [10,17]. These spectral diagrams enabled visualization of variations in VOC profiles across the grain-filling stages.

Compared with the reference spectrum (Xinliang 169 at 5 DAA), the difference spectrum (Figure 2C) showed that red signals indicated higher VOC concentrations and blue signals indicated lower concentrations, revealing distinct dynamic changes during grain filling. Samples from the early stage (5–10 DAA) exhibited dense, diverse peaks, reflecting active primary metabolism, including fatty acid oxidation and amino acid catabolism. The middle stage (20–30 DAA) showed reduced peak intensity, coinciding with rapid starch and protein accumulation, during which metabolic resources are redirected toward the synthesis of storage reserves. The late stage (40 DAA) showed a renewed increase in signal intensity, indicating the accumulation of lipid oxidation product intermediates as grain moisture declines and the environment becomes more concentrated.

A total of 80 volatile compounds were detected by GC-IMS analysis (Table S1), including 10 alcohols, 11 esters, 11 ketones, 19 aldehydes, 6 acids, 7 furans, 10 heterocycles, and 6 others. An additional 26 signals corresponding to dimeric IMS forms of these compounds were not counted as independent volatiles. As shown in Figure 2D, the VOC types detected in the two wheat cultivars were similar, but their relative contents differed significantly. Aldehydes were most prevalent (30.42–42.11%), followed by ketones (15.39–27.75%), esters (8.31–11.37%), alcohols (7.63–16.76%), acids (3.48–5.19%), furans (4.74–7.46%), heterocyclic compounds (3.56–7.70%), and others (3.03–6.79%) of the total VOCs. Aldehydes are major contributors to cereal aroma [10,30], with (*E*)-2-hexenal-M, 3-methylbutanal, and hexanal derivatives showing the highest peak intensities.

The VOC composition of cereals is primarily governed by genetic factors, yet environmental inputs and post-harvest handling further modify the observed aroma profiles [29]. Cultivar-specific differences in VOC accumulation were also observed. XL169 showed advantages in ester accumulation and in the retention of heterocyclic compounds, suggesting a more persistent, stable fruity-green flavor profile. In contrast, XL607 accumulated higher levels of ketones and alcohols during the mid-to-late stages of grain filling. These results suggest that VOC accumulation patterns are genotype-dependent, likely reflecting differences in lipid metabolism and stress responses [31].

A fingerprint gallery plot (Figure 2E) and hierarchical clustering analysis further resolved stage- and genotype-specific accumulation patterns. Notably, some compounds produced multiple spots corresponding to monomers (M), dimers (D), or trimers (T), depending on their concentration and chemical nature [32]. Brighter colors indicate higher content, while darker colors indicate lower content. Based on the visualized GC-IMS signal intensities, the fingerprint spectra were divided into six regions (A–F). Regions A and B were enriched at the early stage (5–10 DAA) and dominated by short-chain aldehydes and ketones, primary metabolites derived from the initial mobilization of fatty acids and amino acids. Region C peaked during the middle stage (10–20 DAA) and comprised medium-chain aldehydes and formates, reflecting a metabolic transition as grain filling accelerated. Regions D and E accumulated predominantly in the late stage (30–40 DAA), particularly in XL169, and were characterized by esters and pyrazines—compounds that contribute substantially to mature, roasted, and fruity flavor notes. Notably, Region E also showed substantial accumulation in the early stage, suggesting a bimodal distribution and indicating that certain volatiles may play dual roles: as primary metabolites during early development and as secondary metabolites contributing to mature flavor. Region F maintained stable accumulation throughout grain filling and contained persistent aroma-active compounds that likely contribute to the baseline flavor profile of wheat.

#### 3.2.2. OPLS-DA and VIP Analysis by GC-IMS

**Figure 3:**
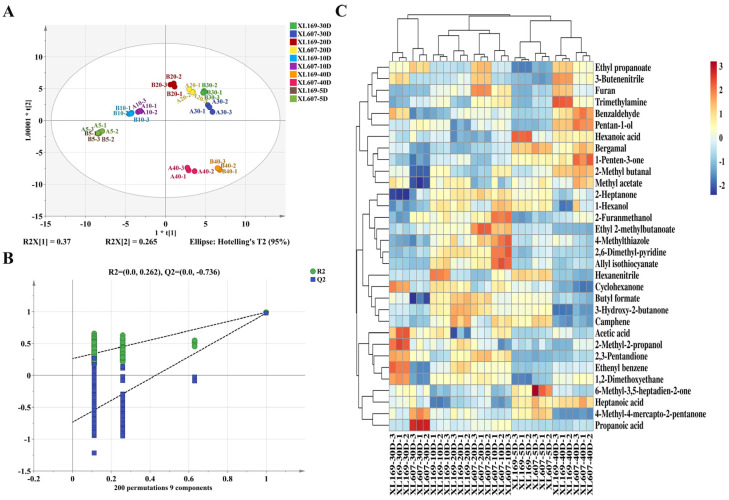
Profiling of volatile compounds across grain-filling stages using HS-GC-IMS. (**A**): OPLS-DA score plot, with A representing XL607 and B representing XL169. (**B**): Permutation test (n = 200) for model validation. (**C**): Cluster heatmap based on VIP values greater than 1; data were autoscaled using a cube-root transformation before visualization. Rows represent individual samples, and columns represent volatile compounds. Hierarchical clustering was applied to both samples and variables.

OPLS-DA was performed to further visualize group discrimination and identify the volatile compounds that contribute most to the separation. As shown in Figure 3A, the model exhibited high goodness-of-fit and predictive ability, with cumulative parameters of R^2^X = 0.991, R^2^Y = 0.974, and Q^2^ = 0.943. A permutation test with 200 random permutations was conducted to assess the potential risk of overfitting (Figure 3B). The results showed that all permuted R^2^ and Q^2^ values were lower than the original values, with the Q^2^ regression line intercept at –0.72 (and the R^2^ intercept at 0.257), supporting the model’s predictive capability. However, given the limited sample size relative to the number of variables, the OPLS-DA results should be interpreted with caution. As Ström et al. [33] note, reducing sample sizes can inflate R^2^ and Q^2^ values, and permutations conducted after variable selection may falsely suggest significant models. Therefore, the OPLS-DA results are presented primarily as exploratory visualizations and for candidate marker prioritization.

Additionally, variable importance in projection (VIP) scores were used to further identify and distinguish wheat samples across grain-filling stages. The VIP value reflects a variable’s contribution to the model’s overall fit and classification performance. The higher a variable’s VIP value, the more important it is in model construction [34]. Generally, variables with VIP values greater than 1 are considered particularly important to the model. A hierarchical clustering heatmap was generated from differential volatile compounds with VIP > 1 to compare normalized metabolite abundances between XL169 and XL607 across five grain-filling stages (Figure 3C). Clear genotypic divergence emerged during late grain filling (30–40 D), and genotype-dependent accumulation patterns of alcohols, ketones, and organic acids were the primary drivers of inter-cultivar metabolic differences. 

### 3.3. HS-SPME-GC-MS Analysis of VOCs in Two Wheat Varieties

#### 3.3.1. VOCs Identified by GC-MS

**Figure 4:**
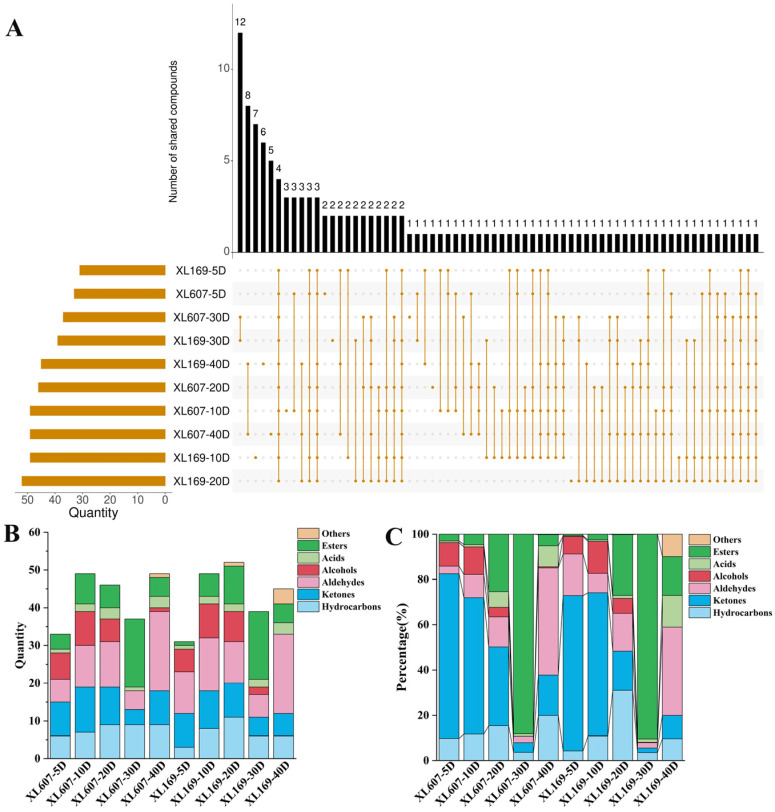
VOC profiles detected by HS-SPME-GC-MS in wheat groups at different grain-filling stages. (**A**): Upset diagram; the horizontal bar chart shows the number of volatile substances in each group; the vertical bar chart shows the number of shared compounds for each intersection combination; the lower dot matrix indicates the grouping combination of co-occurring volatiles. (**B**): Categories and numbers of VOCs. (**C**): Relative percentages of volatile compounds; the stacked bars show the percentage contribution of each compound class to the total volatile compounds detected in each wheat type.

HS-SPME-GC-MS analysis was conducted to complement the GC-IMS dataset. Upset plot analysis revealed cultivar-divergent temporal dynamics of differential metabolites between wheat varieties XL169 and XL607 (Figure 4A). XL169 exhibited stage-specific unique metabolites at 10 DAA (7), 30 DAA (2), and 40 DAA (7), whereas XL607 showed relatively fewer unique metabolites across stages. Notably, shared metabolite pools between the two varieties increased substantially towards later stages, peaking at 30 DAA (12 VOCs) and then at 40 DAA (8 VOCs), indicating a progressive metabolic convergence during mid-to-late grain maturation. A total of 125 volatile compounds were identified (Table S2, Figure 4B), including 17 hydrocarbons, 19 ketones, 31 aldehydes, 18 alcohols, 5 acids, 31 esters, and 4 other compounds. The high proportions of ketones and aldehydes in wheat grains align with previous studies on cereal volatiles [18,35,36], suggesting involvement of lipid oxidation pathways. The Upset diagram (Figure 4A) clearly shows the common and unique substances for each wheat group. Four VOCs, including pentacosane, heneicosane, 6,10,14-trimethyl-2-pentadecanone, and benzaldehyde, were consistently detected across all wheat samples.

The relative proportions of VOC classes differed markedly between cultivars and across stages (Figure 4B). VOCs in both wheat cultivars consisted mainly of hydrocarbons (2.94–22.75%), aldehydes (1.96–31.84%), ketones (1.61–65.59%), acids (0.21–9.33%), alcohols (0.0–11.27%), and esters (0.49–74.87%). Significant variability in volatile compounds has been observed across cereal varieties, with aldehydes, ketones, and esters contributing most to the overall composition of the samples [27]. Aldehydes, the primary VOCs in grains, are formed via the Maillard reaction, Strecker degradation, and lipid peroxidation [35]. They show an overall upward trend, with a low point at 30 DAA (1.96%) and a peak at 40 DAA (23.97%). Alcohols, key flavor components formed by the oxidation of unsaturated fatty acids [36], decreased progressively from 5 to 10 DAA onward.

Ketones were dominant in the early stages (5 DAA: 47.56%; 10 DAA: 45.16%), but decreased sharply to 12.65% at 20 DAA and remained low thereafter. Ketones may originate from fatty acid oxidation, amino acid degradation, alcohol oxidation, and ester decomposition [37]. They typically exhibit woody and burnt aromas, and their scent intensity increases with carbon chain length. Conversely, ester levels increased dramatically from 2.01% at 10 DAA to 19.71% at 20 DAA, peaking at 73.82% at 30 DAA, indicating a metabolic shift from ketone- to ester-dominated volatiles during mid-to late grain filling. Esters are typically formed by esterifying alcohols with free fatty acids and impart sweet, fruity aromas to cereal products [38]. Acids peaked at 40 DAA, likely derived from lipid hydrolysis as membrane integrity declines during late maturation. Acids undergo esterification, forming abundant esters along with trace amounts of aldehydes, ketones, and other characteristic flavor compounds [39]. Alkanes and alkenes typically have fragrant, sweet-smelling aromas, but they also have a higher flavor threshold, so they contribute little to the flavor of wheat flour even at high concentrations [40]. Nevertheless, they may modulate the volatility and flavor-imparting properties of other volatile compounds.

Consistent with previous studies showing that genotype significantly affects aldehyde and ester levels in wheat [41], the present study likewise observed cultivar-specific differences. At 40 DAA, XL169 exhibited significantly higher levels of esters, phenols, and furans, suggesting greater Maillard reaction potential and a more persistent fruity-green flavor profile. In contrast, XL607 exhibited higher levels of hydrocarbons, ketones, aldehydes, and alcohols, indicating more active lipid oxidation. Collectively, ketones and alcohols dominated the early stage (5–10 DAA), esters the middle stage (20–30 DAA), and hydrocarbons, aldehydes, and acids the late stage (40 DAA). These compositional variations contributed to the distinct flavor profiles of the two wheat cultivars.

#### 3.3.2. Multivariate Statistical Analysis of Different Grain-Filling Stages in Two Wheat Cultivars by HS-SPME-GC-MS

**Figure 5:**
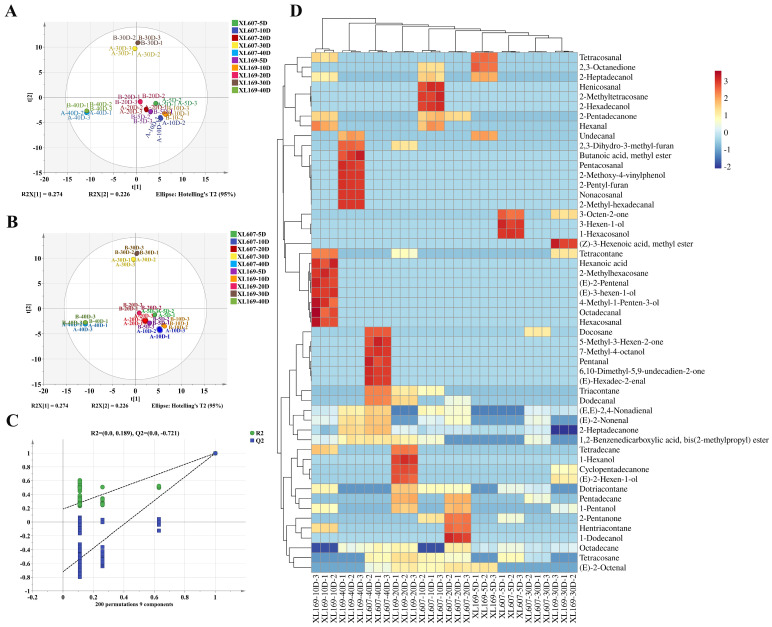
VOC profile of wheat at different filling stages using HS-SPME-GC-MS. (**A**): PCA score plot of VOCs detected in wheat samples. (**B**): OPLS-DA score plot. (**C**): Permutation test (*n* = 200) for model validation. (**D**) Cluster heatmap based on VIP values > 1; data were autoscaled (cube-root transformation) before visualization. Rows represent individual samples, and columns represent volatile compounds. Hierarchical clustering was applied to both samples and variables.

To further discriminate among volatile profiles across cultivars and grain-filling stages, PCA and OPLS-DA models were constructed to assess the discriminatory power of GC-MS-derived VOC profiles. As shown in Figure 5A, based on dynamic changes in VOCs, the entire wheat stage can be divided into three stages: stage I (5–20 DAA), stage II (30 DAA), and stage III (40 DAA), indicating that the later grain-filling stage has a significant impact on volatile components. At 5, 10, and 20 DAA, the samples cluster together, indicating similar VOC profiles. OPLS-DA was used to identify differentially expressed VOCs (Figure 5B). The model showed excellent predictive performance, and its validity was further confirmed by 200 permutation tests (Figure 5C), with R^2^ and Q^2^ ranging from 0.0 to 0.189 and 0.0 to −0.721, respectively. The Q^2^ regression line intercept was below zero, indicating no overfitting. Previous studies have demonstrated that multivariate statistical analyses can effectively discriminate among wheat varieties from different origins and genetic backgrounds [11,42].

VIP scores were calculated to identify the most discriminative VOCs. A total of 53 candidate differential VOCs with VIP > 1 were identified (Figure 5D), comprising 15 aldehydes, 3 esters, 12 alcohols, 8 ketones, 11 alkanes, 1 acid, and 3 other compounds. For cultivar differentiation, several compounds showed marked differences in abundance between XL169 and XL607. Specifically, aldehydes, including hexanal, nonanal, and dodecanal, and esters, including hexadecanoic acid ethyl ester and octadecanoic acid ethyl ester, exhibited differential abundance between the two cultivars. Additionally, 2-pentylfuran and certain C6 alcohols (1-hexanol, 3-hexen-1-ol) were relatively more abundant in XL169, whereas 2,3-octanedione and 3-octen-2-one were more abundant in XL607.

Temporal variation showed that most volatiles remained at low abundance during the early grain-filling stage (5 DAA and 10 DAA), with only small amounts of short-chain ketones and alcohols accumulating. During the late grain-filling stage (20–40 DAA), unsaturated aldehydes, enols, and furans were significantly upregulated. Compounds with similar structures exhibited consistent accumulation trends: long-chain alkanes and saturated aldehydes showed identical variation patterns; enols, enals, and furans were clustered together and were specifically produced in late grain-filling XL607 samples. However, these structural groupings reflect chemical classification and statistical discrimination rather than aroma activity. Their contributions to wheat aroma require further validation using ROAV analysis.

### 3.4. Comparative Analysis of HS-SPME-GC-MS and HS-GC-IMS Results

To clarify the overlap and differences between the two analytical platforms, a comparison of Tables S1 and S2 shows that 80 compounds were detected by GC-IMS, 125 VOCs by GC-MS, and 205 by the combination of both methods. Of these, 19 compounds were consistently detected by both methods, including 3-hydroxy-2-butanone, 2,3-pentandione, 2,3-butanedione, nonanal, benzaldehyde, phenylacetaldehyde, (*E*)-2-octenal, 2-hexenal, heptanal, octanal, (*E*)-2-heptenal, (*E*)-2-pentenal, 1-hexanol, (*Z*)-2-penten-1-ol, 1-octen-3-ol, pentan-1-ol, hexanoic acid, acetic acid, and 2-pentyl-furan. This overlapping set of compounds, many of which are known odor-active volatiles, constitutes a robust core of wheat odor markers that can be reliably detected across analytical platforms.

HS-GC-IMS offers simple operation, high sensitivity, high resolution, high efficiency, and VOC visualization, though its qualitative capability is constrained by limited spectral library coverage [17]. In contrast, HS-SPME-GC-MS offers high separation efficiency, strong identification capability, and high sensitivity, but it cannot distinguish structural isomers, is time-consuming, and requires relatively complex operational procedures and extraction conditions [29,43]. GC-IMS uniquely detected short-chain aldehydes and ketones (e.g., 2-methylpropanal, 3-methylbutanal, 2-butanone) and multiple monomer-dimer pairs, underscoring its sensitivity to small-molecule volatiles and to concentration-dependent clustering. Conversely, GC-MS uniquely identified long-chain fatty acid esters (e.g., hexadecanoic acid methyl ester, octadecanoic acid ethyl ester) and alkanes (e.g., tetracosane), capturing lipid-derived and waxy aroma components of mature wheat grains. However, in complex wheat matrices, co-elution may occur on both platforms, potentially causing ion suppression or enhancement. For low-abundance compounds and stage-specific markers, relative quantification is less precise because of lower S/N ratios. Accordingly, the data obtained represent relative abundances and are intended for comparative rather than absolute quantification.

By contrast, electronic noses and portable sensors return only holistic fingerprints without compound identification [8]; direct GC-MS has 5000-fold lower sensitivity than SPME-based methods [44]; and non-targeted metabolomics faces inherent limitations in spectral convolution, sensitivity, annotation coverage, and reproducibility [45]. Therefore, for mechanistic studies of grain filling that require compound-resolved volatile dynamics, our dual-platform approach—despite longer analysis times and higher costs—is preferred over faster but information-limited alternatives. Collectively, the complementary use of these two platforms provided more comprehensive coverage of the wheat volatile metabolome, encompassing both small-molecule markers and broader chemical diversity.

### 3.5. Screening and Analysis of Key Volatile Compounds

**Figure 6:**
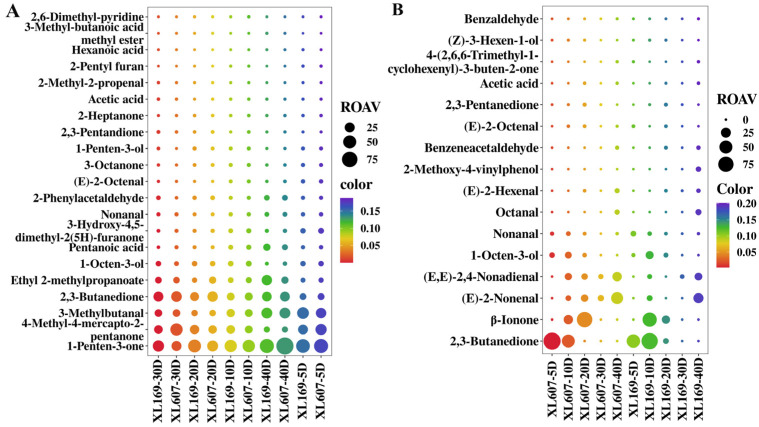
Bubble plot of ROAVs across the grain-filling stage for two wheat cultivars. (**A**): ROAV calculated by GC-IMS; (**B**): ROAV calculated by GC-MS. Bubble size is proportional to each compound’s ROAV.

Large numbers of VOCs were detected in the sample, but only a few key odor compounds contribute substantially to food flavor. To preliminarily prioritize these VOCs by their theoretical odor significance, the relative odor activity value (ROAV) was calculated as a semi-quantitative reference. As shown in Table S3 and Figure 6, the ROAV of compounds was determined by normalizing to 1-penten-3-one (GC-IMS) and 2,3-butanedione (GC-MS). VOCs with ROAV ≥ 1 were considered high-priority candidate odorants, whereas those with 0.1 ≤ ROAV < 1 were regarded as potential modifiers of the wheat odor. Across grain-filling stages, 37 VOCs with ROAV ≥ 0.1 were identified using the two platforms, including 18 priority VOCs with ROAV ≥ 1: 1-penten-3-one,4-methyl-4-mercapto-2-pentanone, 3-methylbutanal, 2,3-butanedione, ethyl 2-methylpropanoate, 1-octen-3-ol, pentanoic acid, 3-hydroxy-4,5-dimethyl-2(5H)-furanone, nonanal, 2-phenylacetaldehyde, β-Ionone, (*E*)-2-nonenal, (*E*, *E*)-2,4-nonadienal, 1-octen-3-ol, octanal, (*E*)-2-hexenal, 2-methoxy-4-vinylphenol, and benzeneacetaldehyde. Among the 18 VOCs identified, 3-methylbutanal and benzeneacetaldehyde mainly arise from the Strecker degradation of leucine and phenylalanine, respectively. 3-Methylbutanal is associated with malty and chocolate-like notes and is the primary contributor to wheat germ aroma [46].

Lipid-derived volatiles, including nonanal, 2-nonenal, (*E*)-2,4-nonadienal, (*E*, *E*)-2,4-nonadienal, 1-penten-3-one, 1-octen-3-ol, and pentanoic acid, which are primarily generated from lipoxygenase catalysis and autoxidation of endogenous polyunsaturated fatty acids (linoleic acid and linolenic acid) in wheat bran and flour [10]. After lipid peroxidation, unstable fatty acid hydroperoxides undergo homolytic cleavage to yield the saturated straight-chain aldehyde nonanal, the unsaturated monoenal 2-nonenal, and the conjugated diene aldehydes (*E*)-2,4-nonadienal and (*E*, *E*)-2,4-nonadienal. Short-chain alkenone 1-penten-3-one and unsaturated alcohol 1-octen-3-ol are also formed via primary hydroperoxide decomposition [47]. This pattern aligns with the progressive accumulation of fatty acid substrates during grain maturation and is consistent with previous findings that these compounds serve as markers of wheat storage time and cultivar discrimination [17,31,48]. The presence of 3-hydroxy-4,5-dimethyl-2(5H)-furanone and 2,3-butanedione is likely attributable to the extraction temperature (80 °C), which may have induced Maillard reactions in the samples. Certain compounds exhibited pronounced cultivar-specific accumulation patterns that may serve as genotypic markers.

Ethyl 2-methylpropanoate, an ester-type volatile biomarker, accumulated preferentially in XL169, consistent with previous findings that this compound distinguishes heritage landraces from modern varieties [6]. Similarly, β-Ionone showed significantly higher levels in XL607 during the mid-to-late stage, supporting its role as a discriminant marker for wheat species differentiation [15,18]. Additionally, 4-methyl-4-mercapto-2-pentanone, a sulfur-containing ketone with meaty and roasted odors, was detected only in XL169 at 40 DAA, suggesting a unique capacity for sulfur-volatile biosynthesis during late grain maturation.

The aroma profile evolved in a clear, stage-dependent manner. Early grain filling (5–10 DAA) was dominated by inferred grassy-green, malty, and vegetable notes, as indicated by literature-derived odor descriptors, with an overall decrease in total ROAV. Middle grain filling (20–30 DAA) featured enhanced buttery, woody, and fruity notes, along with aldehyde accumulation. During late grain filling (30–40 DAA), a mature profile dominated by waxy, fatty, and grain-like aromas emerged, reflecting the continuous generation of lipid oxidation products that define the basic characteristics of wheat flavor. Wheat aroma formation is a complex process involving genetic factors, lipid degradation, amino acids and proteins, microbial metabolism, and their interactions [29]. Cultivar-specific odor differences further support the regulatory role of genotype. Based on the literature on odor descriptors, XL169 exhibited inferred grassy-green, malty, and fatty, grain-like notes, whereas XL607 exhibited creamy, buttery, and sweet-fruity, woody notes.

In addition, odor thresholds are highly matrix-dependent, so ROAVs provide a semi-quantitative prioritization rather than an absolute measure of sensory impact. Further studies are needed to determine the contributions of key volatiles to the overall aroma properties and intensity of wheat through absolute quantification, aroma recombination, and omission testing.

### 3.6. Mantel Test for the Correlation Between VOCs and Quality Traits

**Figure 7:**
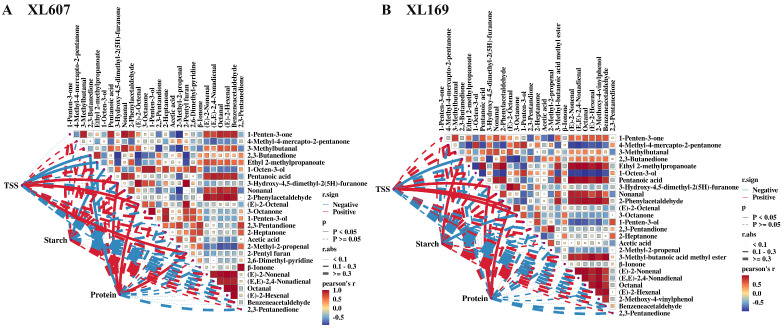
Mantel test of high-priority candidate odorants with quality traits across grain-filling stages.

Mantel analysis is widely used in food science to explore relationships between quality traits and VOCs. A Mantel test was conducted to explore associations between high-priority candidate odorants (ROAV≥1) and quality traits (starch, protein, and TSS) across grain-filling stages (Figure 7). For XL607, TSS was positively correlated with 3-hydroxy-4,5-dimethyl-2(5H)-furanone (r = 0.931, *p* = 0.021), 2-pentyl furan (r = 0.950, *p* = 0.013), (*E*)-2-octenal (r = 0.901, *p* = 0.037) and negatively correlated with ethyl 2-methylpropanoate (r = −0.892, *p* = 0.044) and 2,3-butanedione (r = −0.954, *p* = 0.011). Protein content was positively correlated with 3-hydroxy-4,5-dimethyl-2(5H)-furanone (r = 0.926, *p* = 0.024), (E)-2-octenal (r = 0.986, *p* = 0.002), 2-pentyl furan (r = 0.905, *p* = 0.035) and negatively correlated with 2,3-butanedione (r = −0.965, *p* = 0.008). Starch was also negatively correlated with 1-octen-3-ol (r = −0.890, *p* = 0.043). For XL169, more pronounced correlation patterns were observed. TSS was positively correlated with 3-hydroxy-4,5-dimethyl-2(5H)-furanone (r = 0.925, *p* = 0.025), (*E*)-2-octenal (r = 0.918, *p* = 0.028), 3-octanone (r = 0.973, *p* = 0.005) and negatively correlated with 2,3-butanedione (r = −0.967, *p* = 0.007). Protein content was positively correlated with (*E*)-2-Octenal (r = 0.973, *p* = 0.005) and with 3-hydroxy-4,5-dimethyl-2(5H)-furanone (r = 0.956, *p* = 0.011). Starch content was negatively correlated with 3-octanone (r = −0.928, *p* = 0.023) and positively correlated with 2,3-butanedione (r = 0.901, *p* = 0.037).

3-Hydroxy-4,5-dimethyl-2(5H)-furanone and (*E*)-2-octenal were positively correlated with protein and TSS in both varieties. 3-Hydroxy-4,5-dimethyl-2(5H)-furanone formation depends on TSS, amino acid composition, and thermal processing conditions [1]. (E)-2-Octenal is a volatile unsaturated aldehyde primarily formed via lipoxygenase (LOX)-mediated oxidative degradation of polyunsaturated fatty acids, particularly linoleic and linolenic acids, and is widely documented in various cereal matrices [49]. 2,3-Butanedione was negatively correlated with protein and TSS across both varieties. 2,3-Butanedione forms primarily through non-enzymatic oxidative decarboxylation of α-acetolactate during microbial fermentation and via the Maillard reaction between reducing sugars and amino acids during thermal processing. Its presence in wheat grains at late-filling stages likely reflects progressive sugar–amino acid interactions or residual microbial activity, both of which contribute to the characteristic creamy note of mature grain. However, data gathered in this study cannot precisely pinpoint the exact mechanism.

These association patterns align with prior correlative findings linking VOCs in wheat grains to quality traits, such as protein content [2]. However, as methodological studies of the Mantel test note, correlations between distance matrices should be interpreted cautiously because they may reflect underlying structures rather than direct causal relationships. Accordingly, the present findings offer preliminary associative evidence rather than mechanistic proof, and further targeted experiments are needed to establish causal links between specific quality traits and volatile formation.

## 4. Conclusions

This study investigated dynamic changes in quality traits and VOC profiles in two wheat cultivars contrasting in gluten strength (XL169, medium-strong gluten; XL607, medium-weak gluten) across five grain-filling stages. The following main conclusions can be drawn: (1) Quality traits changed significantly during grain filling. From 5 to 40 DAA, TKW, kernel hardness, and starch content increased significantly in both cultivars, whereas moisture and TSS decreased progressively. (2) The grain-filling stage significantly influences wheat VOC profiles. Based on literature-derived odor descriptors, odor characteristics shifted from grassy and malty notes (early stage, 5–10 DAA) to buttery, woody, and fruity notes (middle stage, 20–30 DAA), and finally to waxy, fatty, and grain-like notes (late stage, 40 DAA). (3) Nineteen volatile compounds were identified as potential priority markers using complementary HS-GC-IMS and HS-SPME-GC-MS, together with OPLS-DA (VIP > 1) and ROAV (≥1) screening. These include 1-penten-3-one, 2,3-butanedione, nonanal, and 3-methylbutanal. (4) The two cultivars exhibited distinct VOC profiles. Based on literature-derived odor descriptors, XL169 was associated with grassy-green, malty, and fatty grain-like notes, whereas XL607 was associated with creamy, buttery, and sweet-fruity woody notes. (5) Quality traits (starch, TSS, and protein) are correlated with certain VOCs (e.g., 2,3-butanedione, 3-hydroxy-4,5-dimethyl-2(5H)-furanone, (*E*)-2-octenal).

Together, these findings document dynamic VOC changes during wheat grain filling and provide a preliminary prioritization of candidate odorants associated with developmental stages and quality traits. Future studies could validate the proposed stage-specific and quality-associated VOC markers by using a broader panel of cultivars and integrating multi-omics technologies to explore the underlying metabolic pathways. Such validations would provide a basis for understanding the metabolic pathways that underlie aroma development in wheat.

## Data Availability

The original contributions presented in this study are included in the article/Supplementary Materials. Further inquiries can be directed to the corresponding authors.

## Supplementary Materials

The following supporting information can be downloaded at: https://www.mdpi.com/article/10.3390/foods15152639/s1, three supplementary tables are provided for reference: Table S1: Volatile compounds detected in two wheat cultivars by HS-GC-IMS; Table S2: Identification of volatile compounds across different grain-filling stages in two wheat cultivars by HS-SPME-GC-MS; Table S3: Relative odor activity values (ROAV) of volatile compounds.
